# Combination of serological biomarkers and clinical features to predict mucosal healing in Crohn’s disease: a multicenter cohort study

**DOI:** 10.1186/s12876-022-02304-y

**Published:** 2022-05-10

**Authors:** Nana Tang, Han Chen, Ruidong Chen, Wen Tang, Hongjie Zhang

**Affiliations:** 1 Department of Gastroenterology, The First Affiliated Hospital of Nanjing Medical University, 300 Guangzhou Road, Nanjing, China; 2 Department of Gastroenterology, The Second Affiliated Hospital of Soochow University, 1055 Sanxiang Road, Suzhou, China

**Keywords:** Crohn’s disease, Mucosal healing, Nomogram, PLR, Endoscopic

## Abstract

**Purpose:**

Mucosal healing (MH) has become the treatment goal of patients with Crohn’s disease (CD). This study aims to develop a noninvasive and reliable clinical tool for individual evaluation of mucosal healing in patients with Crohn’s disease.

**Methods:**

A multicenter retrospective cohort was established. Clinical and serological variables were collected. Separate risk factors were incorporated into a binary logistic regression model. A primary model and a simple model were established, respectively. The model performance was evaluated with C-index, sensitivity, specificity, positive predictive value (PPV), negative predictive value (NPV) and accuracy. Internal validation was performed in patients with small intestinal lesions.

**Results:**

A total of 348 consecutive patients diagnosed with CD who underwent endoscopic examination and review after treatment from January 2010 to June 2021 were composed in the derivation cohort, and 112 patients with small intestinal lesions were included in the validation cohort. The following variables were independently associated with the MH and were subsequently included into the primary prediction model: PLR (platelet to lymphocyte ratio), CAR (C-reactive protein to albumin ratio), ESR (erythrocyte sedimentation rate), HBI (Harvey-Bradshaw Index) score and infliximab treatment. The simple model only included factors of PLR, CAR and ESR. The primary model performed better than the simple one in C-index (87.5% vs. 83.0%, *p* = 0.004). There was no statistical significance between these two models in sensitivity (70.43% vs. 62.61%, *p* = 0.467), specificity (87.12% vs. 80.69%, *p* = 0.448), PPV (72.97% vs. 61.54%, *p* = 0.292), NPV (85.65% vs. 81.39%, *p* = 0.614), and accuracy (81.61% vs. 74.71%, *p* = 0.303). The primary model had good calibration and high levels of explained variation and discrimination in validation cohort.

**Conclusions:**

This model can be used to predict MH in post-treatment patients with CD. It can also be used as an indication of endoscopic surveillance to evaluate mucosal healing in patients with CD after treatment.

**Supplementary Information:**

The online version contains supplementary material available at 10.1186/s12876-022-02304-y.

## Background

Crohn’s disease (CD) is a life-long and progressive inflammatory disease with symptoms evolving in a remitting and relapsing manner [1]. Most patients develop bowel damage and disability including strictures, fistulas, abscesses and so on in several years after diagnosis, resulting in surgery [2]. Current therapeutic strategies in CD aim for deep and prolonged remission, with the goal of preventing complications and therefore improve prognosis as well as quality of life [3]. Treatment strategies aimed solely at resolution of clinical symptoms does not eliminate long-term bowel damage in patients with CD [4].

Mucosal healing (MH) is confirmed lead to a lower rate of relapse, hospitalization and surgical resection [5]. In recent years, MH is preferred over clinical remission as straightforward goal of clinical treatment in CD [6]. Endoscopy is still the golden standard for evaluation of disease activity. However, frequently endoscopy for disease monitoring in long-term follow-up is limited by considerations of invasiveness, high cost and patient acceptance. Alternative noninvasive methods are necessary for assessment of CD-related mucosal inflammation. [7, 8]

It is reported that clinical characteristics such as mild clinical manifestation and early introduction of biologicals associated with MH in patients with CD [9–11]. In addition, some systemic inflammatory markers obtained from the serological examination including neutrophil to lymphocyte ratio (NLR) and platelet to lymphocyte ratio (PLR) [12], C-reactive protein to Albumin ratio (CAR) [13], combination of fecal calprotectin (FC), erythrocyte sedimentation rate (ESR), C-reactive protein (CRP) and Albumin (ALB) [14] have been explored as diagnostic and predictive indicators of CD. Clinically, there is still lack of accurate instrument with high sensitivity and specificity using serological parameters and clinical characteristics to predict MH in CD.

In this study, we aimed to address the predictive role of serum inflammatory index and clinical features in patients with CD who diagnosed and treated in two tertiary hospitals in China. We analyzed the pre-treatment and post-treatment data individually and explored their relationship with MH after treatment including Corticosteroids, Immunomodulators or Infliximab in patients with CD. Subsequently, we used hematological data with or without clinical features to construct assessment models for MH prediction. Model with superior performance is recommended for clinical use.

## Methods

### Patients and data source

This was a retrospective, multi-center observational cohort study of consecutive patients with CD from Inflammatory Bowel Disease Center of The First Affiliated Hospital of Nanjing Medical University and the Second Affiliated Hospital of Soochow University, China, between 2010 and 2021. Diagnoses of CD were determined according to standard clinical, laboratory, radiological, endoscopic, and histopathologic findings [15]. Data regarding patients’ demographics, laboratory values and endoscopic characteristics were retrospectively reviewed through hospital medical database records and endoscopic image system. Harvey-Bradshaw Index (HBI) consists of five descriptors: general well-being, abdominal pain, number of liquid stools for the previous day, abdominal mass and complications. [16].

Inclusion criteria were listed as follows: (1) Patients underwent at least twice endoscopic procedures and serological examination both pre-treatment and post-treatment during the study period. (2) Corticosteroids had been discontinued for more than 12 weeks. Exclusion criteria: (1) Acute or chronic infections during the inspection; (2) Previous medical history of hematologic or rheumatic autoimmune disease; (3) Acute or chronic renal failure, heart diseases, cirrhosis or cancer; (4) A previous history of taking aspirin or warfarin; (5) Missing complete blood count, ALB, ESR or CRP data. (6) Any other conditions that affect the blood routine results or inflammatory markers. (7) Patients treated with novel biologicals including adalimumab (ADA), ustekinumab (UST) or vedolizumab (VDZ). In the present study, serological indicators were used to construct mucosal healing model of CD patients. So, we ruled out the effects of drugs on blood cells including corticosteroids, aspirin and warfarin. The clinical, endoscopic features and laboratory data of the study population are summarized in Tables 1 and 2.

**Table 1:** Demographic and clinical characteristics of MH and Non-MH patients

	Mucosal healing n (%)	Non-mucosal healing n (%)	*p* Value
Number of patients	115 (33)	233 (67)	
Gender			0.120
Male	85 (35.7)	153 (64.3)	
Female	30 (27.3)	80 (72.7)	
Smoking			0.701
Non-smoker	99 (32.7)	204 (67.3)	
Smoker	16 (35.6)	29 (64.4)	
Family history of IBD			1.000
No	113 (33.1)	228 (66.9)	
Yes	2 (28.6)	5 (71.4)	
Surgical history			0.799
No	95 (32.8)	195 (67.2)	
Yes	20 (34.5)	38 (65.5)	
Disease location			0.055
L1 Ileal	41 (36.6)	71 (63.4)	
L2 Colonic	13 (20.3)	51 (79.7)	
L3 Ileocolonic	61 (35.5)	111 (64.5)	
Upper digestive tract involved			0.412
No	92 (32.4)	192 (67.6)	
Yes	24 (37.5)	40 (62.5)	
Stenosis			**0.005**
No	97 (37.2)	164 (62.8)	
Yes	18 (20.7)	69 (79.3)	
Penetrating			0.074
No	225 (68.0)	106 (32.0)	
Yes	8 (47.1)	9 (52.9)	
Perianal lesion			0.626
No	59 (31.9)	126 (68.1)	
Yes	56 (34.4)	107 (65.6)	
*Medication treatment*			
Corticosteroids			0.953
No	98 (33.1)	198 (66.9)	
Yes	17 (32.7)	35 (67.3)	
Immunomodulators			0.72
No	95 (32.6)	196 (67.4)	
Yes	20 (35.1)	37 (64.9)	
Infliximab			** < 0.001**
No	39 (17.4)	185 (82.6)	
Yes	76 (61.3)	48 (38.7)	

MH, mucosal healing; Bold indicates *P* < 0.05

**Table 2:** Logistic regression for hematological parameters evaluation of MH

Blood tests	Pre-treatment			Post-treatment		
	Non-MH M (Q)	MH M (Q)	*P* value	Non-MH M (Q)	MH M (Q)	*p* Value
WBC (10^9^/L)	7.02 (3.43)	7.23 (3.60)	0.276	6.27 (2.70)	5.86 (2.23)	0.205
NE (10^9^/L)	5.19 (2.82)	5.44 (2.74)	0.985	4.17 (2.18)	3.39 (1.75)	** < 0.001**
MO (10^9^/L)	0.54 (0.35)	0.52 (0.28)	0.790	0.97 (0.26)	0.42 (0.20)	**0.001**
EO (10^9^/L)	0.20 (0.12)	0.26 (0.17)	**0.021**	0.21 (0.13)	0.12 (0.08)	0.209
BA (10^9^/L)	0.03 (0.02)	0.03 (0.02)	0.687	0.04 (0.03)	0.02 (0.02)	0.239
HGB (g/L)	117.9 (34.8)	121.8 (31.0)	0.161	125.1 (34)	134.1 (23)	** < 0.001**
HCT (%)	42.29 (9.33)	37.29 (9.1)	0.582	38.5 (8.45)	40.4 (6.7)	**0.003**
PLT (10^9^/L)	304.9 (128)	300.57 (155)	0.943	275.9 (120)	230.3 (79)	** < 0.001**
CRP (mg/L)	25.87 (31.7)	24.65 (30.76)	0.086	17.2 (17.33)	3.41 (2.15)	** < 0.001**
ESR (mm/h)	29.52 (34.0)	27.09 (35.0)	0.139	25.9 (30.4)	9.54 (11.0)	** < 0.001**
NLR	4.41 (2.45)	6.52 (2.56)	0.112	3.69 (2.29)	2.03 (1.07)	** < 0.001**
MLR	0.42 (0.30)	0.53 (0.24)	**0.020**	0.67 (0.25)	0.25 (0.15)	** < 0.001**
PLR	242 (154.2)	475.8 (148)	**0.015**	228.6 (124)	137.1 (77.4)	** < 0.001**
CAR	0.80 (0.98)	0.72(0.82)	**0.044**	0.49 (0.48)	0.09 (0.06)	** < 0.001**
PAR	8.89 (5.24)	8.20(5.39)	0.209	7.46 (3.44)	5.52 (2.11)	** < 0.001**

MH, mucosal healing; WBC, White Blood Cell; NE, Neutrophils; MO, Monocyte; EO, Eosinophils; BA, Basophils; HGB, Hemoglobin; HCT, hematocrit; PLT, platelet; CRP, C reactive protein; ESR, erythrocyte sedimentation rate; NLR, Neutrophil–Lymphocyte Ratio; MLR, Monocyte-Lymphocyte Ratio; PLR, Platelet-Lymphocyte Ratio; CAR, C reactive protein-Albumin Ratio; PAR, Platelet- Albumin Ratio; Bold indicates *P* < 0.05

Ethical approval for the study was approved by Clinical Research Ethics Committee of The First Affiliated Hospital of Nanjing Medical University (ref: 2021-SR-235), in compliance with the Declaration of Helsinki. All patients in the study gave their informed consent for reviewing their clinical data.

### Blood assessment and endoscopic documentation

Baseline blood values had been collected at the time of CD diagnosis when patients were admitted to hospital before administration of any treatment. Post-treatment hematology was completed within one week of the patient's endoscopic review. Venous blood specimens were drawn into sterile standard tubes containing ethylene diamine tetraacetic acid (EDTA) as an anticoagulant and evaluated within 1 h after venipuncture using a Beckman Coulter UniCel DxH800 hematology analyzer. The Beckman Coulter UniCel® DxH 800 was used for analyzing ESR and routine blood markers including White Blood Cell (WBC), neutrophils (NE), monocytes (MO), lymphocytes (LY), Eosinophils (EO), Basophilic (BA), Hemoglobin (HGB), platelet (PLT) and hematocrit (HCT). The Beckman Coulter AU5800 Clinical Chemistry Analyzer was used for assessing ALB and CRP. Inflammatory markers of NLR, Monocyte-to-Lymphocyte Ratio (MLR), PLR, CRP-ALB Ratio (CAR) and Platelet-ALB Ratio (PAR) were calculated subsequently.

Patients underwent at least twice endoscopic examination during the study, before treatment and approximately one year after treatment (10–14 months), respectively. Mucosal status was assessed by double-balloon enteroscopy or colonoscopy according to the lesion site. Endoscopic procedures were performed with the standard protocol and the static endoscopic images were reassessed retrospectively by two experienced gastroenterologists. MH was defined as a mucosal activity of gastrointestinal tract as remission or mild inflammatory activity, without ulcer [16, 17]. Disease phenotype was established according to Montreal Classification. [18, 19].

### Statistical analysis

The statistical analyses were performed by using SPSS 26.0 software (SPSS, Chicago, IL, USA). Normality test were applied by Shapiro–Wilk test. Data with normal distribution are presented as mean with Standard deviation (SD), and data with non-normal distribution are presented as median with Interquartile Range (Q). The *t* test (2-tailed) was applied for data with normal distribution while Mann–Whitney *U* test were performed in data with abnormal distribution. Chi-square tests or Fisher's exact test were used to compare the nonparametric categorical data between groups.

Univariate and multivariant analyses were applied in SPSS. R software (version 3.3.2) was used to build the nomogram and evaluation of model performance (“rms” package). Parameters inclusive of the interaction terms and of clinical significance were included in a full multivariate model subsequently. The model performance was evaluated with C-index, sensitivity, specificity, positive predictive value (PPV), negative predictive value (NPV) and accuracy. To simplify the logistic regression results and create a practical tool, the coefficients derived from the multivariate analysis were used as weights to elaborate a nomogram, which facilitates the practical application of the model for evaluating probability of MH expected for a given patient. Internal validation was performed in patients with small intestinal lesions. *p* Value less than 0.05 was considered statistically significant.

## Results

### Demographic and clinical characteristics of patients

A total of 348 patients with CD were enrolled into present study and 115 patients achieved MH. Baseline demographic and clinical characteristics are shown in Additional file 4: Table S1. The median diagnosis age of the included patients was 28.0 years (IQR: 21–39 years) and median disease course of all the individuals was 12 months (IQR: 4–36 months). Median HBI score of the patients was 7 (IQR: 5–9). Shapiro–Wilk test showed that data of diagnosis age, disease course and HBI score were with abnormal distributions (all *p* < 0.001). Thus, Mann–Whitney *U* test was performed and identified that levels of HBI (*p* = 0.006) and age at diagnosis (*p* = 0.003) were significantly associate with MH, while the disease course was not associated with MH (*p* = 0.893). In addition, chi-square analyze showed that patients without lumen stenosis (*p* = 0.005) and treatment with infliximab (*p* < 0.001) are associated with MH. (Table 1).

### Pre-treatment hematological parameters and mucosal healing

Pre-treatment laboratory blood parameters of patients with CD were summarized in Table 2. Shapiro–Wilk test showed that all the data of blood test were with abnormal distributions (all *p* < 0.001). Thus Mann–Whitney *U* test was performed and identified that the levels of Eosinophils (*p* = 0.021), MLR (*p* = 0.020), PLR (*p* = 0.015) and CAR (*p* = 0.044) were significantly associate with MH (Table 2). Furthermore, these significant factors were selected to further perform multivariate regression analysis and only PLR was associated with MH after treatment (*p* = 0.037). However, the ROC curve analysis showed that AUC of PLR were only 0.58 (95% CI 0.515–0.644, *p* = 0.015) and specificity was only 0.313, lacking of clinical application significance.

### Post-treatment hematological parameters and mucosal healing

Shapiro–Wilk test showed that all the data of blood test after treatment of 54 weeks were with abnormal distributions (all *p* < 0.001). Mann–Whitney *U* test identified that the levels of NE (*p* < 0.001), MO (*p* = 0.001), HGB (*p* < 0.001), HCT (*p* = 0.003), PLT (*p* < 0.001), CRP (*p* < 0.001), ESR (*p* < 0.001), NLR (*p* < 0.001), MLR (*p* < 0.001), PLR (*p* < 0.001), CAR (*p* < 0.001), PAR (*p* < 0.001) were significantly associate with MH (Table 2). In the multivariate regression, we identified the following three variables as the independently associated factors with MH: PLR, CAR and ESR.

### Model establishment

We established two models: a simple model and a primary model. The simple model (model-1) only contains serum biomarkers including PLR, CAR and ESR. The primary model (model-2) was consisted of the following variables: PLR, CAR, ESR, HBI score and treatment with infliximab. Variables included in the simple and primary models are showed in Table 3.

**Table 3:** Multivariate logistic regression of models for Mucosal healing evaluation

	Simple model (model-1)		Primary model (model-2)	
	OR [95%CI]	*P* value	OR [95% CI]	*p* Value
HGB	0.996 [0.971–1.021]	0.754	0.986 [0.952–1.022]	0.437
HCT	0.968 [0.877–1.068]	0.519	0.978 [0.861–1.112]	0.734
NE	0.909 [0.754–1.096]	0.317	0.847 [0.676–1.060]	0.147
MO	0.950 [0.756–1.194]	0.661	0.848 [0.120–5.984]	0.848
CAR	0.022 [0.002–0.219]	**0.001**	0.036 [0.004–0.320]	**0.003**
PLR	0.993 [0.989–0.997]	**0.001**	0.995 [0.990–0.999]	**0.014**
ESR	0.955 [0.928–0.982]	**0.001**	0.951 [0.922–0.981]	**0.002**
Age	NA	NA	0.993 [0.967–1.021]	0.682
HBI	NA	NA	0.907 [0.824–0.999]	**0.047**
Stenosis	NA	NA	0.599 [0.289–1.241]	0.168
Infliximab	NA	NA	6.346 [3.324–12.117]	** < 0.001**

HGB, Hemoglobin; HCT, hematocrit; NE, Neutrophils; MO, Monocyte; CAR, C reactive protein-Albumin Ratio; PLR, Platelet-Lymphocyte Ratio; ESR, erythrocyte sedimentation rate; HBI, Harvey-Bradshaw Index; Bold indicates *P* < 0.05

### Comparisons between simple model and primary model

Diagnostic value was compared between the two models. The golden standard is whether MH has been achieved under endoscopy. Table 4 shows the classification of the two models.

**Table 4:** Comparison of simple model and primary model

Diagnostic Index	Simple model (%, 95% CI)	Primary model (%, 95% CI)	*p* Value
C-index	0.830 (0.79–0.87)	0.875 (0.84–0.91)	0.004*
Sensitivity	62.61% (53.10–71.45%)	70.43% (61.21- 78.58%)	0.467
Specificity	80.69% (75.02–85.55%)	87.12% (82.13- 91.14%)	0.448
PPV	61.54% (54.29–68.31%)	72.97% (65.45–79.37%)	0.292
NPV	81.39% (77.39–84.81%)	85.65% (81.76- 88.83%)	0.614
Accuracy	74.71% (69.80–79.20%)	81.61% (77.13–85.54%)	0.303

^*^Statistically significant with a p-value less than 0.05

Simple Model: model constructed from PLR, CAR and ESR; Primary Model: model constructed from PLR, CAR, ESR, HBI and IFX treatment; PPV, positive predictive value; NPV, negative predictive value; Bold indicates *P* < 0.05

**Fig. 1:**
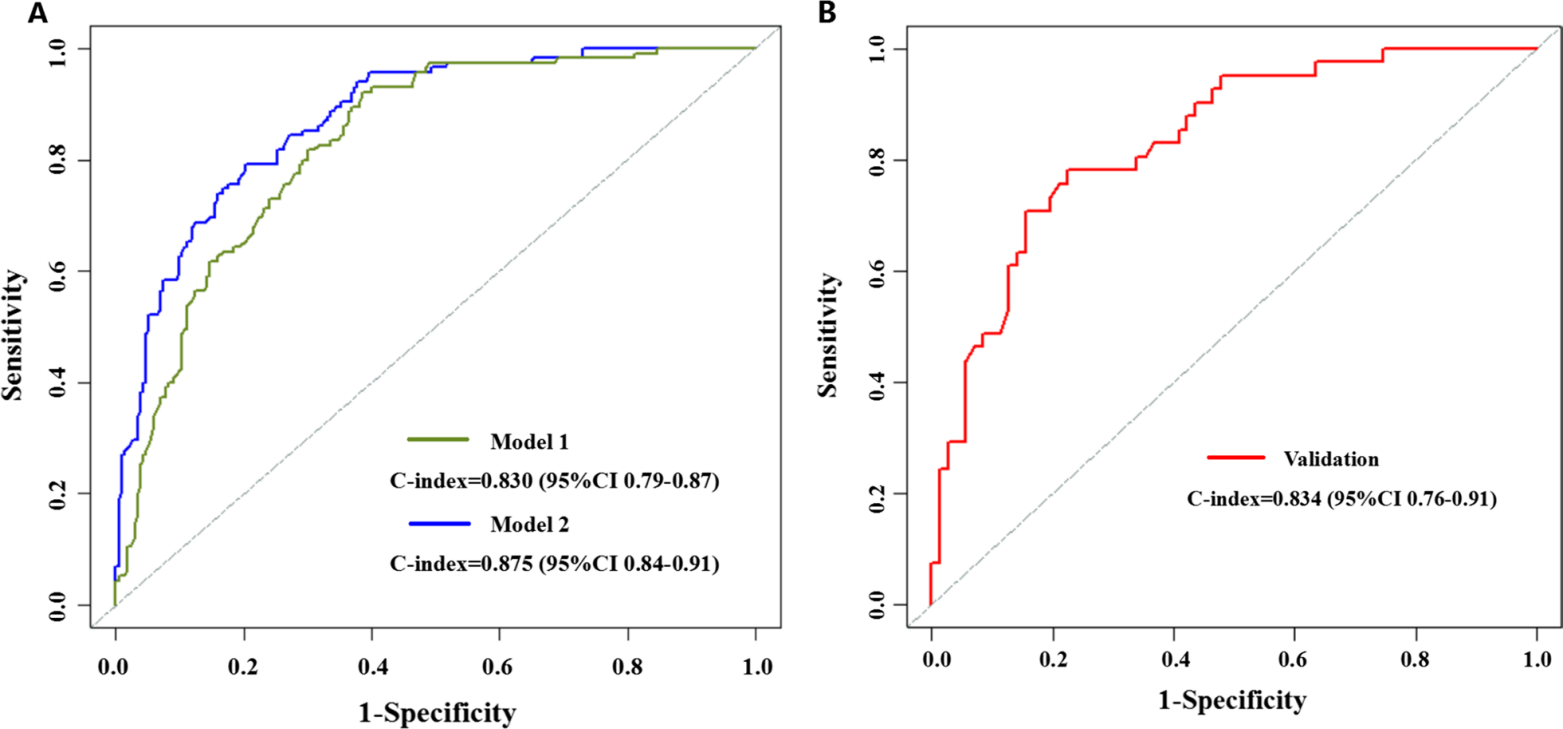
ROC curve analysis of simple model (model-1) and primary model (model-2) in training group (**A**); ROC curve analysis of primary model in validation group (**B**)

The C-index of simple model was 0.830 (95% CI 0.79–0.87, *p* < 0.001) (Fig. 1A). The sensitivity and specificity were 0.626 and 0.807, respectively (Table.4). Primary model showed a perfect capacity for predicting MH, with a C-index of 0.875 (95% CI 0.84–0.91, *p* < 0.001) (Fig. 1A). Sensitivity of primary model was 0.704 and specificity was 0.871 (Table 4). According to DeLong’s test, there is significant difference of C-index between primary model and simple model (Z = 2.8519, *p* = 0.0043). Primary model was superior to simple model in C-index (87.5% vs. 83.0%, *p* = 0.004). There was no statistical significance between primary model and simple model in sensitivity (70.43% vs. 62.61%, *p* = 0.467), specificity (87.12% vs. 80.69%, *p* = 0.448), PPV (72.97% vs. 61.54%, *p* = 0.292), NPV (85.65% vs. 81.39%, *p* = 0.614), and accuracy (81.61% vs. 74.71%, *p* = 0.303) (Table 4).

### Evaluation and validation of the primary nomogram

**Fig. 2:**
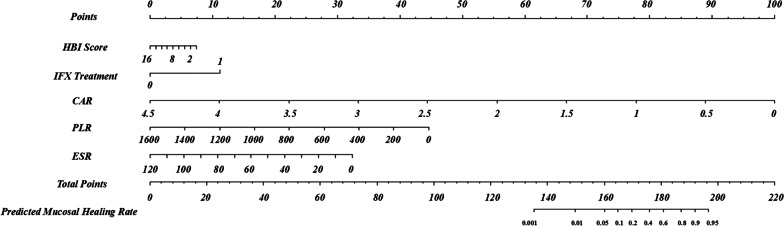
Nomogram for evaluation of MH rate in a given patient, constructed using as weights the coefficients derived from multivariate analysis. To calculate the probability of MH, we first obtained the value of each evaluator by drawing a vertical line straight upward from that factor to the points’ axis, then summed the points achieved for each factor and located this sum on the total points’ axis of the nomogram, where the probability of MH can be located by drawing a vertical line downward. MH, mucosal healing

**Fig. 3:**
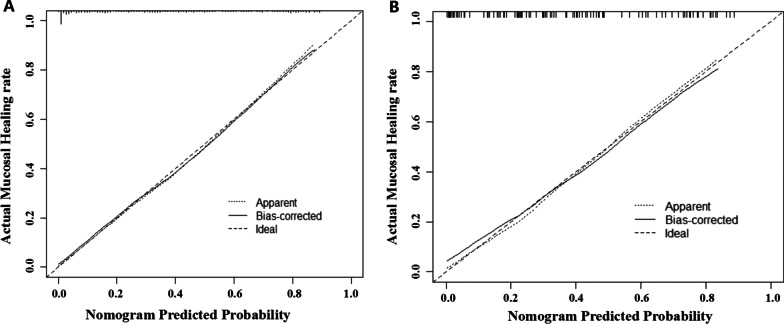
Calibration curves for primary model in (**A**) training cohort and (**B**) validation cohort. The x-axis represents the predicted MH while y-axis represents actual MH rate. The 45-degree dotted lines represent a perfect prediction. The solid line represents the performance of the evaluation models. The closer solid line fits to the dotted line, the better accuracy of the model shows. MH, mucosal healing

A nomogram was established based on the variables in the primary model (Fig. 2). Model performance was evaluated by discrimination and calibration. This model had a high C-index (0.88) as mentioned above. The calibration curve also showed satisfactory performance (Fig. 3A). The internal validation was performed in 112 patients with small bowel involvement. After validation, the C-index of the model was 0.834 (95% CI 0.76–0.91, *p* < 0.001) (Fig. 1B). The calibration curve in validation group is shown in Fig. 3B. The internal validation also performed good in discrimination and calibration.

## Discussion

In the present study, the most important findings include: (1) Serum biomarkers such as PLR, CAR and ESR after approximately 1 year treatment was independently associated with MH. (2) Patients with lower HBI score and the use of infliximab are more likely to achieve MH. Based on this, we developed prediction model with the above significant variables to evaluate MH in patients with CD.

The routine blood test is the most fundamental and accessible examination, which has long been proposed as an essential assistant tool for disease assessment [20]. In our study, there was no statistical significance between pre-treatment serum inflammatory indexes and MH. Thus, we were unable to develop a pre-treatment model. However, in the post-treatment data, we found the combination use of PLR, CAR and ESR can effectively evaluate MH in patients with CD. Platelet count can be affected by cytokines released in acute inflammation. Thrombocytosis and high ESR level are common feature of acute inflammation. Lymphocytes is the basic component of the adaptive and innate immune system. It is demonstrated that PLR increased significantly in endoscopically active ulcerative colitis [21]. CRP is the most widely used serological indicators in clinical evaluation of disease activity in CD [22, 23]. Serum ALB is an indicator of nutrition, synthesis rate of which directly affected by the severity of acute infection. CAR was initially used to identify critical patients in emergency ward and predict disease progression in Takayasu arteritis and cancer in recent years [24, 25]. It is reported that CAR is useful biomarker of disease activity and histological activity in CD [26]. Consistent with previous research, we included the above three variables including PLR, CAR and ESR into our model.

Some clinical characteristics can also predict MH in patients with CD. It is reported that early introduction of tumor necrosis factor (TNF) antagonists, particularly in combination with immunosuppressives associate with MH in patients with CD. [27] In our study, patients received infliximab was significantly associate with an increased rate of MH, consistent with previous reports. HBI was derived to simplify calculation of the Crohn's disease activity index (CDAI). We newly found that HBI was associated with MH. In univariate logistic regression analyses, diagnose age and lumen stenosis were found associate with MH in the present data. Unfortunately, neither of them was included in the final multivariate logistic regression model.

A simple model and a primary model were established in the study. The simple model is simpler, easier to operate clinically, and with favorable accuracy. However, taking into account of the C-index and the calibration plots, primary model showed better discrimination ability. Reliable nomogram based on aforementioned factors was constructed and showed excellent evaluation abilities for MH among patients with CD. Parameters in the monogram are easy to obtain, which increases the clinical practicality. This nomogram can predict MH probability in patients with CD after one year of treatment and provide reference for doctors to perform endoscopic review. If the prediction results indicate low probability of MH, doctors could temporarily eliminate endoscopy and adjust treatment regimen, avoiding repeated and unnecessary invasive endoscopy.

Fecal calprotectin (FC) has been widely clarified for the correlation with endoscopically proven CD activity [28–30]. However, FC is still not commonly used in clinical practice because of detection results may vary from different kits of calprotectin. In addition, some researchers pointed out that PPV of FC for MH was not high enough and FC was not sensitive to assess CD activity with small intestine involvement [31, 32]. For these reasons, we did not include FC in present study. Nevertheless, we validated primary model in patients with CD with small intestine involvement, indicating a good evaluation effect.

Our study has some limitations. Firstly, although the patients we included from two tertiary hospitals in Eastern China, the results may not represent the general population of patients with CD. Secondly, this is a retrospective study and novel biologics such as Vedolizumab and Ustekinumab were not included as evaluation item in the model. The present model still could be improved by follow-up study and improved to broaden the scope in evaluation of CD.

In summary, this study provides comprehensive insights into serum inflammatory index and clinical information to evaluate MH after treatment in patients with CD. We conducted a nomogram, providing a portable decision tool for early MH screening and clinical decision of endoscopic review time. More prospective studies in the future are warrant to perform.

## Supplementary Information

**Additional file 1**. **Excel 1**. Patients’ data before treatment.

**Additional file 2**. **Excel 2**. Patients’ data after treatment.

**Additional file 3**. **Excel 3**. General information of the patients.

**Additional file 4**. **Table S1**. Baseline demographic and clinical characteristics of patients

## Data Availability

We share our raw data by providing it in a supplementary file.
