# Supplementary material for: Combination of serological biomarkers and clinical features to predict mucosal healing in Crohn’s disease: a multicenter cohort study
Source: BMC Gastroenterol. 2022 May 10;22:229. doi: 10.1186/s12876-022-02304-y (PMC9088028; doi:10.1186/s12876-022-02304-y)
Supplement: Supplementary file 4 — Additional file 4. Table S1. Baseline demographic and clinical characteristics of patients [file 12876_2022_2304_MOESM4_ESM.docx]

Suppl. Table 1. Baseline demographic and clinical characteristics of patients

|  | n (%) |
| --- | --- |
| Number of patients  Mucosal healing  Non-mucosal healing  Age at diagnosis  A1 < 17 years  A2 17-40 years  A3 > 40 years  Gender  Male  Female | 115(33.0)  233(67.0)  23(6.6)  249(71.6)  76(21.8)  238(68.4)  110(31.6) |
| Disease duration  ≤ 10 months  ＞ 10months  HBI score  < 5  5 - 8  ＞8  Smoking | 141(40.5)  207(59.5)  121(34.8)  130(37.3)  97(27.9) |
| Non-smoker  Smoker | 303(81.7)  45(12.9) |
| Family history of IBD  No  Yes | 341(98.0)  7(2.0) |
| Surgical history  No  Yes | 290(83.3)  58(16.7) |
| Disease location |  |
| L1 Ileal | 112(32.2) |
| L2 Colonic | 64(18.4) |
| L3 Ileocolonic | 172(49.4) |
| Upper digestive tract involved  No  Yes | 284(81.6)  64(18.4) |
| Stenosis |  |
| No  Yes  Penetrating | 261(75)  87(25) |
| No  Yes | 331(95.1)  17(4.9) |
| Perianal lesion |  |
| No | 185(53.2) |
| Yes | 163(46.8) |
| Therapeutic agents |  |
| Corticosteroids  Immunomodulators  Infliximab | 52(14.9)  57(16.4)  124(35.6) |

**Abbreviations:** MH, mucosal healing
